# Malignant peripheral nerve sheath tumor of the ileocecal region: a case report

**DOI:** 10.3389/fonc.2026.1882494

**Published:** 2026-07-03

**Authors:** Yiqian Luo, Yuanyuan Wang, Rentong Liu, Yiwei Cheng, Chengjie Gan, Bing Li

**Affiliations:** Department of Gastrointestinal Surgery, The 967th Hospital of the Joint Logistic Support Force of the Chinese People’s Liberation Army, Dalian, China

**Keywords:** case report, colon, ileocecal region, malignant peripheral nerve sheath tumor, MPNST

## Abstract

**Background:**

Malignant peripheral nerve sheath tumor (MPNST) is a rare and aggressive soft tissue sarcoma that rarely occurs in the gastrointestinal tract. To date, fewer than ten cases of primary colonic MPNST have been reported, and this case may represent the first case report of an MPNST arising in the ileocecal region.

**Case presentation:**

A 62-year-old male patient presented with right lower abdominal pain for two days. A firm, fixed mass approximately 10 cm in diameter was palpable in his right lower abdomen. Auxiliary examinations including abdominal CT and colonoscopy revealed various features suggestive of malignant neoplasm, such as: a large, irregularly shaped tumor with infiltrative growth, accompanied by hemorrhagic erosion and enlarged regional lymph nodes. Although endoscopic biopsy suggested high-grade mucosal intraepithelial neoplasia, clinical suspicion remained strongly focused on a malignant tumor of the ileocecal region. Due to concomitant intestinal obstruction symptoms, the patient underwent laparoscopic radical right hemicolectomy following multidisciplinary consultation. Ultimately, multiple pathological examinations combined with immunohistochemistry confirmed the diagnosis of MPNST of the ileocecal region. Follow-up examination six months later revealed no evidence of recurrence or metastasis.

**Conclusion:**

This case provides novel anatomical and clinical information regarding MPNST, a rare and aggressive neoplasm. Due to its atypical clinical manifestations and overlapping histopathological features with other colonic tumors, preoperative definitive diagnosis is difficult and misdiagnosis is common. A confirmed diagnosis relies on postoperative pathological examination and immunohistochemistry. Surgical resection with confirmation of tumor-free margins represents the preferred therapeutic approach.

## 1 Introduction

Malignant peripheral nerve sheath tumor (MPNST) is a mesenchymal tissue-derived neoplasm that arises from the peripheral nerves and exhibits characteristics of nerve sheath differentiation. It accounts for about 5-10% of all soft tissue sarcomas, and the incidence rate in the general population is 1/100, 000 (1). The common sites of occurrence include the trunk, extremities, head and neck, as well as the paravertebral regions. MPNST involving the gastrointestinal tract are extremely rare, and those located in the colon are even more uncommon. According to the literature, there are fewer than 10 reported cases of primary colonic MPNST (2). Herein, we report a case of primary colonic MPNST. To the best of our knowledge, this represents the first case of MPNST occurring in the ileocecal region. This case presented with ileocecal inflammation as the initial symptom. Definitive diagnosis could not be achieved via colonoscopic biopsy or intraoperative frozen section pathological examination. Eventually, the diagnosis of colonic MPNST was confirmed by paraffin section pathological examination combined with immunohistochemistry.

## 2 Case presentation

A 62-year-old male patient with no specific past medical history or family history of tumors presented to our hospital with intermittent right lower abdominal pain for 2 days. A hard, fixed mass with tenderness, approximately 10 cm in diameter, was palpable in his right lower abdomen. On admission, the patient showed signs of infection: white blood cell count (WBC) was 10.64×10^9^/L, C-reactive protein (CRP) was 107 mg/L, accompanied by mild anemia with hemoglobin (Hb) level of 107 g/L. All tumor markers were within normal limits.Abdominal ultrasonography revealed mixed echo in the ileocecal region with surrounding exudative changes, suggesting an inflammatory process and a neoplastic lesion could not be ruled out. After anti-infective treatment, the patient’s abdominal pain was relieved, and follow-up tests showed normalization of WBC and CRP levels. Abdominal contrast-enhanced computed tomography (CT) indicated a space-occupying lesion in the ascending colon and ileocecal region with adjacent fat infiltration, suggestive of malignant tumor (**Figures 1A, B**); enlarged intra-abdominal and retroperitoneal lymph nodes were noted, which were considered as lymph node metastasis (**Figures 1C, D**). Colonoscopy findings suggested colonic stenosis secondary to colon cancer (**Figure 2**). Endoscopic biopsy revealed high-grade intraepithelial neoplasia of the mucosa, together with inflammatory necrotic tissue consistent with ulcerative changes. Although no malignant tumor cells were identified in the biopsy specimens, the clinical diagnosis was highly suspected to be ileocecal malignant tumor complicated with lymph node metastasis (cT_4_N_2+_M_0_).

**Figure 1:**
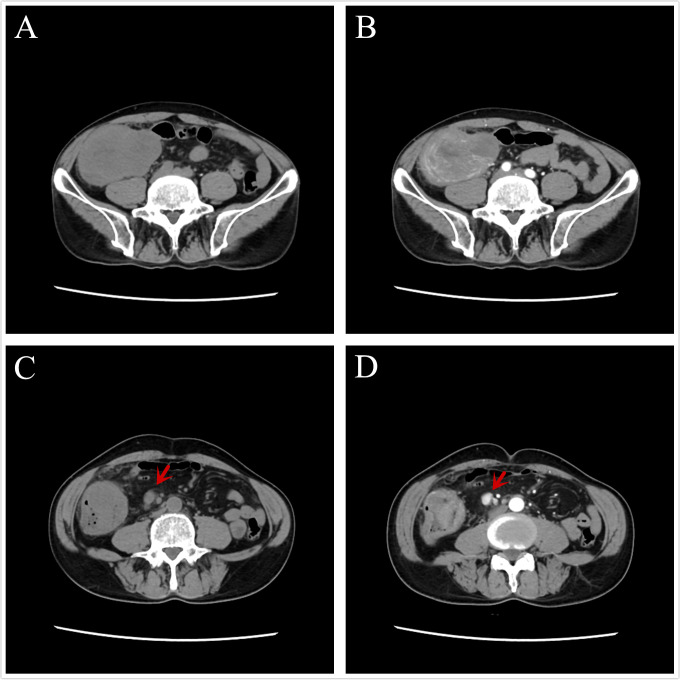
Unenhanced and enhanced CT images after anti-infective treatment. **(A)** A mass-like mixed density lesion is seen in the ascending colon and ileocecal region, with a cross-sectional area of approximately 8.6 × 6.4 cm. **(B)** The lesion shows marked heterogeneous enhancement after contrast administration, with vascular structures traversing the lesion and irregular liquefactive necrosis in the center. Perifocal fat stranding is noted, along with band-like high-density areas with ill-defined margins. **(C, D)** Enlarged intra-abdominal lymph nodes (arrows) show marked enhancement after contrast administration.

**Figure 2:**
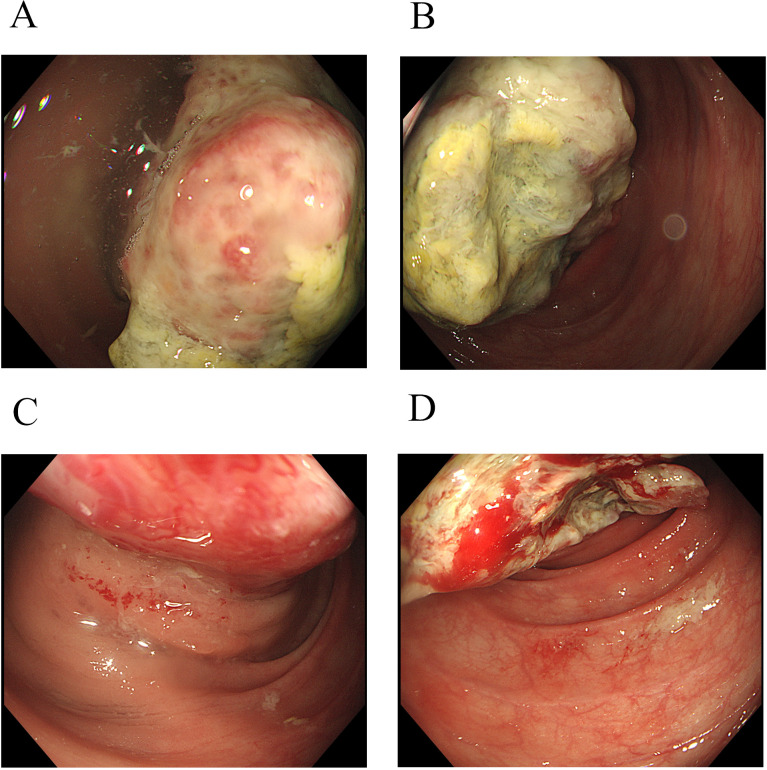
Preoperative colonoscopic images **(A, B)** A huge irregular mass with a rough and uneven surface occupies the intestinal lumen, resulting in luminal stenosis that prevents passage of the endoscope. **(C, D)** The mass has a broad base, involves approximately half of the intestinal circumference, and is accompanied by hyperemia, erosion, and old hemorrhagic foci.

Given the patient presented with symptoms of incomplete intestinal obstruction, a surgical plan was formulated following a multidisciplinary consultation (MDT): laparoscopic right hemicolectomy with D3 lymphadenectomy. Intraoperatively, the ileocecal tumor was found to have penetrated the colonic serosa and invaded the surrounding greater omentum, with multiple enlarged lymph nodes detected in the perienteric region and mesentery. The intraoperative clinical stage was determined as cT_4_N_2+_M_0_.Intraoperative frozen section analysis revealed a mesenchymal spindle cell tumor with significant cellular pleomorphism, suggestive of a low-grade malignant tumor; a definitive diagnosis was pending paraffin section examination.

Postoperative histopathological examination using hematoxylin-eosin (H&E) staining indicated a mesenchymal spindle cell tumor, initially suggestive of a gastrointestinal stromal tumor (GIST); no tumor metastasis was identified in the lymph nodes (0/23).Results of immunohistochemical staining showed: S100 (+), SOX-10 (+), SMA (-), Caldesmon (-), CD34 (-), CD117 (-), DOG1 (-), Ki-67 (10%) (**Figure 3**). These findings indicated a colonic schwannoma; the tumor was large in volume, ill-defined in some areas, and accompanied by focal active cellular proliferation, Numerous mitoses are visible, with a maximum count of 6 mitoses per mm². Ultimately, combined histopathological examination and immunohistochemistry confirmed a diagnosis of high-grade colonic MPNST (**Figure 4**). Ultimately, a definitive diagnosis of colonic malignant peripheral nerve sheath tumor (MPNST) was established based on the combination of histopathological examination and immunohistochemical results.

**Figure 3:**
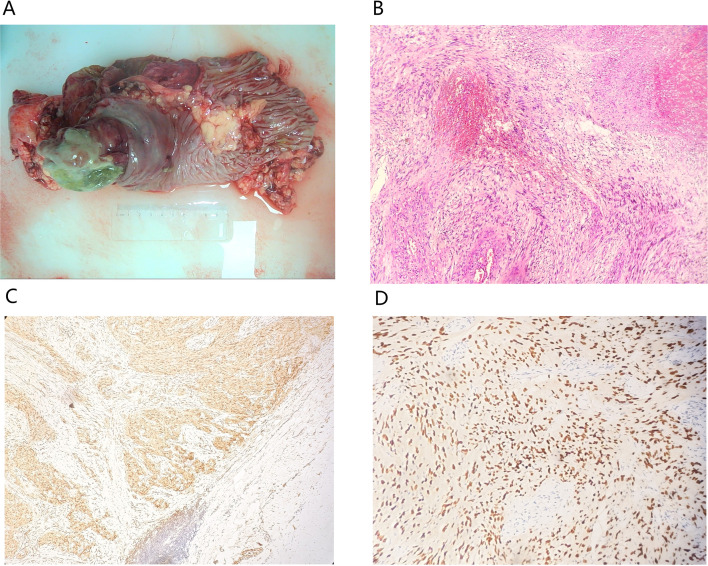
Gross specimen and histopathological findings. **(A)** Gross specimen:A protuberant mass measuring 8.4 × 6.2 × 4.5 cm is observed in the ileocecal region. The cut surface is grayish-white and brittle, with infiltration through the entire intestinal wall. **(B)** Hematoxylin and eosin (H&E) staining of the tumor tissue: Spindle cells are arranged diffusely in a fascicular pattern. **(C)** Immunohistochemical staining showing diffuse S100 positivity. **(D)** Immunohistochemical staining showing diffuse SOX10 positivity.

**Figure 4:**
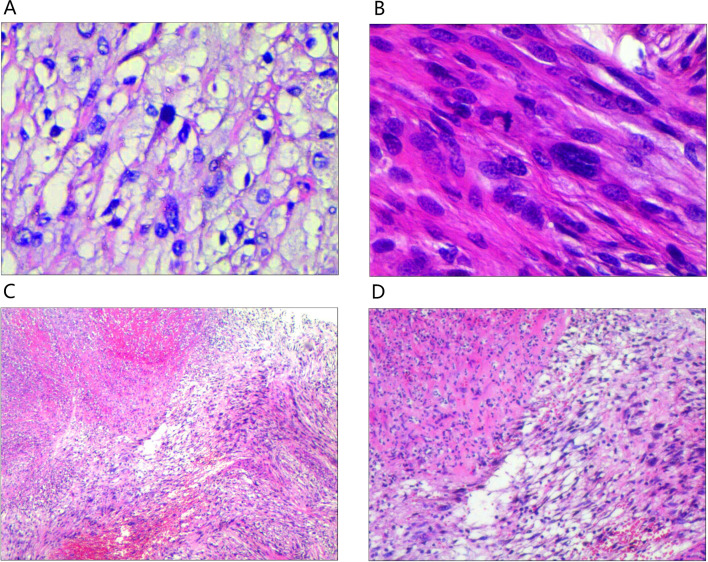
Histopathological findings. **(A, B)** Hematoxylin and eosin (H&E) staining of the tumor tissue: Abundant mitoses were present, up to 6 mitoses/mm² at the hottest spot. **(C, D)** Hematoxylin and eosin (H&E) staining of the tumor tissue: Geographic necrosis is visible in the central area of the tumor.

The patient recovered well postoperatively. Considering that the tumor had achieved R0 resection and was insensitive to chemotherapy, no adjuvant therapy was administered. To date, the patient has been followed up for 6 months with no evidence of recurrence or metastasis.

A timeline summarizing the episode of care is shown in **Table 1**.

**Table 1:** Timeline of episode of care.

Date	Event	Key findings/interventions
2025-05-23	Onset of symptoms	Abdominal pain
2025-05-25	Admission/Lab tests/US	WBC 10.64×10^9^/L, CPR 107mg/L/Ileocecal mixed echo lesion
2025-05-25	anti-infective	abdominal pain relieved
2025-05-27	Contrast-enhanced CT	Ascending colon & ileocecal mass, malignant tumor suspected
2025-05-28	Colonoscopy/Biopsy	Colon carcinoma with bowel stenosis/High-grade intraepithelial neoplasia
2025-05-29	MDT meeting	
2025-05-30	Surgery	Intraop: Ileocecal tumour perforated serosa, omental invasion; Stage: cT_4a_N_2+_M_0_.
2025-05-30	Intraop frozen section	Mesenchymal spindle-cell lesion, severe atypia,
2025-06-04	postoperative pathology	Mesenchymal spindle-cell lesion, GIST?
2025-06-06	Immunohistochemistry	Colonic MPNST
2025-01-10	Follow-up	No recurrence and no metastasis

## 3 Discussion

MPNST, previously termed “malignant Schwannoma”, is considered a spindle-cell sarcoma arising in cells of neural-crest origin in which fibroblasts are frequently present (3). It is worth noting that MPNST does not arise from the malignant transformation of benign peripheral nerve sheath tumors (schwannomas). Despite the existence of multiple subtypes of schwannomas, they are categorized as grade I tumors according to the classification criteria of the World Health Organization (WHO), with an extremely low risk of malignant transformation (4). Gastrointestinal schwannomas are also relatively rare, with the majority occurring in the stomach. Two single-center studies with relatively large case series have demonstrated that the malignant rates of gastrointestinal schwannomas are approximately 0.4% (1/229) (5) and 2.5% (2/78) (6), respectively. In addition, a systematic literature review focusing on colorectal schwannomas showed that their malignant rate is around 3.1% (3/96) (7). Schwannomas occurring in the small intestine, colorectum, and abdominal cavity have a higher malignant potential compared with those located in the stomach (8). Approximately 40%-50% of MPNST cases are associated with neurofibromatosis type 1 (von Recklinghausen’s disease, NF1) gene mutations, while another 40%-47% are sporadic (9). Malignant transformation of schwannomas represents only a rare, specific type of sporadic MPNST cases. However, colonic MPNST may differ from this pattern. A recent study conducted a comparative analysis of all reported cases of primary colonic MPNST and found that only 1 case (12.5%) was associated with NF1 gene mutation (1). This finding is significantly different from the approximately 50% NF1 mutation rate observed in MPNST at other sites, leading the authors to speculate that primary colonic MPNST may be predominantly a sporadic disease. No NF1-related clinical manifestations or family history were identified in the present case.

In existing literature reports, patients with colonic MPNST do not exhibit a concentrated age of onset, with cases documented in both neonates and the elderly (10). Additionally, there are no characteristic clinical manifestations; most patients present with non-specific symptoms such as abdominal pain, intestinal bleeding, and weight loss. For this reason, preoperative diagnosis of colonic MPNST is relatively challenging and often results in diagnostic delay.

It should be highlighted that although the tumor in this case is large, maximum tumor diameter alone cannot act as a diagnostic criterion for distinguishing benign schwannomas from MPNST. Benign schwannomas can also develop into huge lesions without any malignant pathological features. Clinicians ought not to evaluate the biological behavior of nerve sheath tumors based solely on lesion size. To reach an accurate diagnosis, comprehensive assessment integrating mitotic density, the status of tumor necrosis and a full panel of immunohistochemical markers is mandatory.

Colonic MPNST must be differentiated from malignant colonic gastrointestinal stromal tumors, leiomyosarcoma and other analogous malignancies. Clinical manifestations correlate with the tumor’s location and maximum diameter. Imaging modalities, routine laboratory tests, and even basic histomorphological features cannot reliably distinguish MPNST from other malignant spindle-cell neoplasms. Accordingly, definitive diagnosis requires a comprehensive panel of immunohistochemical testing. This colonic MPNST possesses abundant desmoplastic stroma, rendering its morphology highly mimetic of far more prevalent mesenchymal lesions including gastrointestinal stromal tumors and smooth muscle neoplasms. Furthermore, this case highlights that sole dependence on preoperative workup may result in misdiagnosis as colonic GIST or colonic adenocarcinoma, which would compromise subsequent therapeutic management.

MPNST lacks specific molecular markers. S100 and SOX10, the immunohistochemical markers commonly used to detect neural crest-derived tumors, are not highly specific for MPNST (11). In MPNST, the degree of Schwann cell differentiation is often inadequate and heterogeneous, resulting in an S100 positivity rate of approximately 50–60%. The negativity of the S-100 protein may indicate dedifferentiation of Schwann cells, and S100 negativity may even correlate with higher malignant potential (1). Therefore, immunohistochemical analysis adopts a predominantly exclusionary diagnostic approach: negative staining for CD117 and DOG1 can rule out GISTs; negative staining for desmin and SMA can rule out leiomyoma. The Ki-67 labelling index of most malignant tumors generally exceeds 20%, whereas the index of this ileocecal malignant peripheral nerve sheath tumor is merely 10%. We propose the following explanations for this finding. This colonic MPNST falls into the stroma-rich subtype. Unlike conventional sarcomas composed of densely packed tumor cell sheets, the lesion is partitioned by abundant infiltrating collagen fibers. Though focal tumor cells undergo active mitosis, the high stromal proportion and large overall tumor cellular population dilute the global labelling index, resulting in a low proportion of Ki-67-positive proliferating cells. Ki-67 is only one immunohistochemical marker for MPNST, and tumor grade cannot be assessed solely based on this single indicator. Previous published studies have also reported MPNST cases with low Ki-67 levels (12). Notably, regional tumor necrosis serves as an independent criterion for high-grade classification even in lesions with low proliferative activity. According to the 5th edition of the *WHO Classification of Soft Tissue and Bone Tumors*, tumors with more than 5 mitoses per mm² or concomitant tumor necrosis are classified as high-grade MPNST. The core immunohistochemical markers for MPNST, which should be detected as a combined panel, include S-100, SOX-10, Nestin, p16, p53, p27 and H3K27me3.

Due to the rarity of cases, current understanding of colonic MPNST remains limited, and no consensus has been reached regarding the optimal surgical resection margin and postoperative treatment regimen. The roles of adjuvant radiotherapy and chemotherapy are still unclear (13). Existing treatment recommendations and strategies are likely formulated solely based on experience with this tumor at other anatomical sites. Studies have indicated that MPNST is relatively resistant to conventional chemotherapy and radiotherapy, with response rates generally lower than those of other soft tissue sarcomas (14). Therefore, adjuvant therapy is usually administered only in unresectable cases or those with positive resection margins (15). For localized MPNST, complete tumor resection with negative margins is currently the standard treatment.

MPNST is associated with a poor prognosis, with a 5-year survival rate of only 35% to 60% (3), and it tends to develop distant metastasis, most commonly in the lung. A multicenter cohort study on local recurrence of MPNST demonstrated that among 507 patients, the local recurrence rate was 28%. Independent risk factors for local recurrence included high-grade tumor, microscopically positive resection margins, and large tumor size (>5 cm) (16). Primary colonic MPNST carries an even worse prognosis, with a recurrence rate of nearly 40% (3/8) (2).

The patient was informed about the rarity of colonic MPNST and the lack of standardized treatment protocols. After thorough discussion with the multidisciplinary team, the patient expressed a strong preference for radical surgical resection over adjuvant chemotherapy or radiotherapy, given the uncertain benefit and potential toxicity. The patient prioritized quality of life and wished to avoid prolonged hospitalization. Postoperatively, the patient reported satisfactory recovery and remained under close surveillance. In the absence of high-level evidence for ultra-rare sarcomas such as colonic MPNST, incorporating patient values and preferences into shared decision-making is essential.

We present an extremely rare case of primary colonic MPNST. Due to its non-specific clinical symptoms and morphological similarities to more common mesenchymal tumors, preoperative diagnosis is challenging, and definitive diagnosis requires a combination of postoperative pathological examination and immunohistochemical analysis. Unfortunately, further genetic testing was not performed at the patient’s request, although such testing could have helped guide more effective therapeutic strategies. The standard treatment consists of complete surgical resection with negative margins. Long-term follow-up is essential given the tumor’s propensity for late recurrence and metastasis. This case enriches the limited yet expanding body of literature concerning this rare disease. It also carries considerable educational reference value and can serve as a reference for gastroenterologists encountering intestinal spindle-cell lesions of undetermined nature in routine clinical practice.

## Data Availability

The original contributions presented in the study are included in the article/supplementary material. Further inquiries can be directed to the corresponding author.
